# Association between the systemic immune-inflammation index and the outcome of liver fibrosis in patients with chronic hepatitis C

**DOI:** 10.3389/fmed.2024.1486503

**Published:** 2024-11-26

**Authors:** Yuanji Ma, Jiayi Wang, Lingyao Du, Hong Tang

**Affiliations:** Center of Infectious Diseases, West China Hospital of Sichuan University, Chengdu, China

**Keywords:** chronic hepatitis C, liver fibrosis, risk factor, systemic immune-inflammation index, outcome

## Abstract

**Background:**

Risk factors that influence the outcome of patients with chronic hepatitis C (CHC) are not fully understood. The systemic immune-inflammatory index (SII) is an independent prognostic factor for multiple diseases. However, the impact of the SII on the outcome of liver fibrosis is unclear.

**Methods:**

This prospective real-world study enrolled patients with CHC treated with sofosbuvir/velpatasvir. Logistic regression models were used to investigate the relationship between the SII and the outcome of liver fibrosis in treatment-naive patients. Liver fibrosis was assessed using aspartate aminotransferase-to-platelet ratio index (APRI) and fibrosis-4 index (FIB-4).

**Results:**

Of the 288 participants, the SII was 238.2 (153.0–358.2). The non-improved outcomes of liver fibrosis assessed with APRI (non-improved APRI) and FIB-4 (non-improved FIB-4) were 83.0 and 87.5%, respectively. Adjusted models showed that the SII was positively associated with non-improved APRI (adjusted OR (95% CI): 1.013 (1.009–1.017), *p* < 0.001) and FIB-4 (adjusted OR (95% CI): 1.004 (1.001–1.007), *p* = 0.012). Similarly, a higher SII was associated with a higher risk of non-improved APRI (adjusted OR (95% CI): 13.53 (5.60–32.68), *p* < 0.001) and FIB-4 (adjusted OR (95% CI): 5.69 (2.17–14.90), *p* < 0.001). The association with non-improved APRI was much more remarkable in patients with alanine aminotransferase <2 ULN, and the association with non-improved FIB-4 was remarkable in patients aged <50 years. Multiple imputation analyses confirmed the robustness of these results.

**Conclusion:**

Our findings suggested that the SII was positively associated with non-improved outcomes of liver fibrosis in patients with CHC. These results need to be validated in large-scale prospective cohorts.

## Introduction

Hepatitis C virus (HCV) infection remains a global public health issue. A certain proportion of patients with chronic HCV infection may progress to liver cirrhosis or hepatocellular carcinoma (HCC) asymptomatically (1). Fortunately, proper administration of directly acting antivirals (DAAs) can eliminate viruses in over 95% of patients, regardless of liver stiffness (2). However, disease deterioration did not appear to stop after viral elimination. Our previous study reported that HCC events and progression of liver disease did occur in patients after sustained virologic response (SVR), although at a low rate (3). Cirrhosis is a high-risk factor for HCC in patients with chronic hepatitis C (CHC) (4, 5), even after DAA treatment for HCV (5). Surveillance is needed; however, the risk factors that influence the clinical outcomes of patients with CHC are not fully understood. Hepatic stellate cells (HSCs) are central drivers of fibrosis. Therefore, host factors influencing its activation contribute to fibrogenesis. As the inflammatory immune microenvironment plays an important role in HSC activation (6), related indicators may help reflect or predict prognosis. An easy-to-operate indicator for both local immune response and systemic inflammation, the systemic immune-inflammatory index (SII), was first reported in 2014 and is now widely used (7). SII is calculated using peripheral lymphocyte (LYM), neutrophil (NEU), and platelet (PLT) counts (8) and has been identified as an independent prognostic factor for multiple diseases including liver, pancreatic, and colorectal cancers (1, 9, 10), coronary artery disease (11, 12), acute ischemic stroke (13), hypertension (14–16), diabetes mellitus (17–19), kidney stones (20), hepatic steatosis and fibrosis (21, 22), and acute-on-chronic liver failure (23).

For better management of post-HCV-infected individuals, a follow-up strategy consisting of liver function, viral load, liver stiffness, and incidence of HCC is currently under establishment (3). An indicator capable of predicting fibrosis greatly optimizes the follow-up strategy. However, the relationship between SII and the outcome of liver fibrosis in patients with CHC has not been well described. In this study, we conducted a secondary data analysis based on a data set from a prospective real-world study that enrolled patients with CHC treated with a DAA regimen, sofosbuvir/velpatasvir (SOF/VEL), to investigate this relationship.

## Methods

### Study design

A secondary data analysis was conducted based on a data set from a prospective real-world study that enrolled patients with CHC treated with SOF/VEL. The relationship between the SII and the outcome of liver fibrosis in treatment-naive patients was assessed in this study. This real-world study was approved by the Biomedical Research Ethics Committee of West China Hospital of Sichuan University (2019-008) and was conducted in compliance with the Declaration of Helsinki. Informed consent was obtained from all participants and/or their legal guardians.

### Patients

**Figure 1:**
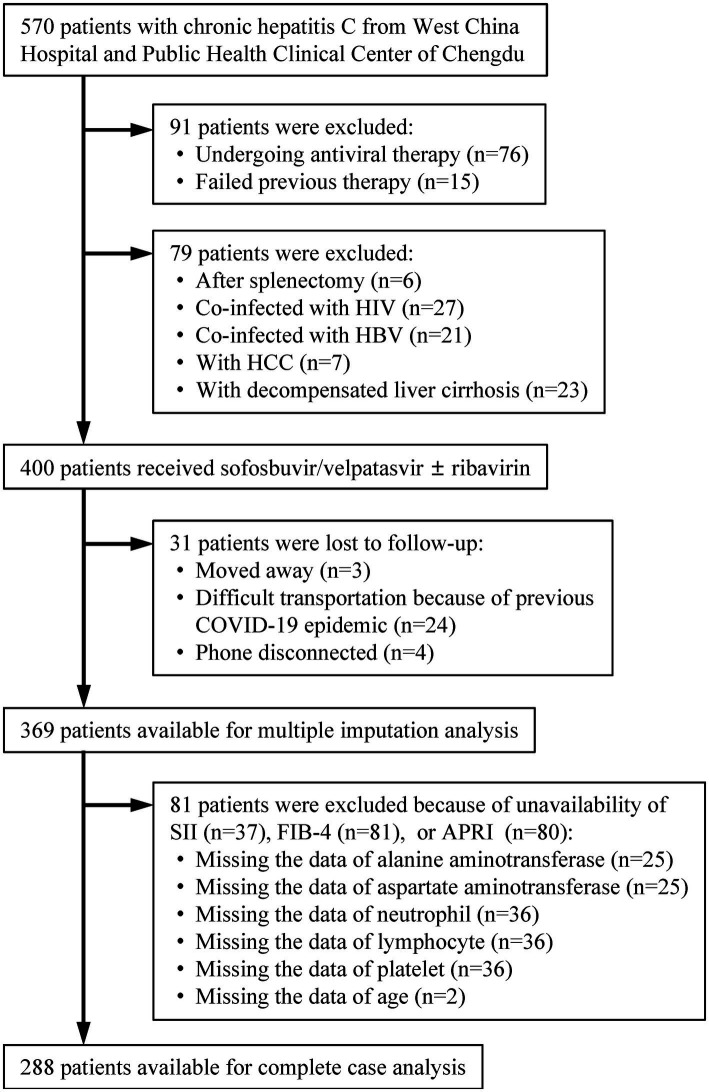
Patients’ selection. HIV, human immunodeficiency virus; HBV, hepatitis B virus; HCC, hepatocellular carcinoma; COVID-19, coronavirus disease 2019; SII, systemic immune-inflammation index; FIB-4, fibrosis-4; APRI, aspartate aminotransferase-to-platelet ratio index.

Patients with CHC were continuously screened at the West China Hospital of Sichuan University and the Public Health Clinical Center of Chengdu from January 2019 to June 2022 (*n* = 570; Figure 1). Patients were excluded if they were undergoing antiviral therapy or had failed previous therapy (*n* = 91). Treatment-naive patients with CHC who underwent splenectomy, were co-infected with human immunodeficiency virus (HIV) or hepatitis B virus (HBV), and had a history of hepatocellular carcinoma (HCC) or decompensated liver cirrhosis were excluded (*n* = 79). Patients who were lost to follow-up and those for whom a sustained virological response at 12 weeks (SVR12) was not available were also excluded (*n* = 31). The remaining patients were eligible for multiple imputation analysis (*N* = 369). After excluding patients due to unavailability of SII, fibrosis-4 index (FIB-4), or aspartate aminotransferase (AST)-to-PLT ratio index (APRI) (*n* = 81), the last patients were available for complete case analysis (*N* = 288).

All treatment-naive CHC patients received SOF/VEL (400/100 mg)-based therapy. The use of ribavirin (RBV) was dependent on the HCV genotype, presence of liver cirrhosis, liver function, and hemoglobin level. The treatment duration (12 or 24 weeks) was dependent on the HCV genotype, the presence of liver cirrhosis, and the use of RBV.

### Assessment of liver fibrosis and SII

The diagnosis of liver cirrhosis was based on ultrasonography and/or computed tomography (CT). Liver fibrosis was assessed using the APRI and FIB-4 (24, 25).

The APRI (24), which is composed of AST (upper limit of normal (ULN)) and PLT (10^9^/L), was developed as follows: $\mathrm{APRI}=\frac{AST\left(ULN\right)}{PLT\left(10^{9}/L\right)}\times 100$ .

The FIB-4 (25), which is composed of age (years), PLT (10^9^/L), alanine aminotransferase (ALT) (IU/L), and AST (IU/L), was developed as follows: $\mathrm{FIB}-4=\frac{\mathrm{Age}\;\left(\mathrm{years}\right)\times AST\left(\mathrm{IU}/L\right)}{PLT\left(10^{9}/L\right)\times \sqrt{\mathrm{ALT}\left(\mathrm{IU}/L\right)}}$ .

The SII (7), which is composed of a peripheral NEU, LYM, and PLT, is defined as follows: $SII=PLT\left(10^{9}/L\right)\times \frac{NEU\left(10^{9}/L\right)}{LYM\left(10^{9}/L\right)}\left(\times 10^{9}/L\right)\text{.}$ The SII was stratified into less than or greater than the cutoff value for further analysis.

### Definition of outcomes

SVR12 was defined as HCV RNA level < 15 IU/mL at 12-week post-DAA treatment (PTW12).

Liver fibrosis was assessed using the APRI and FIB-4 at enrollment and PTW12. An APRI >2.0 or a FIB-4 > 3.25 indicates that the patient has developed advanced liver fibrosis (24, 25). The increase of APRI from ≤2.0 at enrollment to >2.0 at PTW12, or FIB-4 from ≤3.25 at enrollment to >3.25 at PTW12 was defined as the progression of liver fibrosis. The decrease of APRI from >2.0 at enrollment to ≤2.0 at PTW12, or FIB-4 from >3.25 at enrollment to ≤3.25 at PTW12 was defined as the improvement of liver fibrosis. The maintenance of the original level of APRI (≤2.0 or > 2.0) or FIB-4 (≤3.25 or > 3.25) was defined as the stabilization of liver fibrosis. The progression or stabilization of liver fibrosis assessed using APRI and FIB-4 was defined as non-improved outcomes of liver fibrosis (non-improved APRI and FIB-4).

### Statistical analysis

Quantitative data were presented as medians (interquartile ranges) and were compared using Mood’s median test. Qualitative data were presented as frequencies (proportions) and compared using the chi-square test. The optimal cutoff values of the SII (stratified SII) for non-improved APRI and FIB-4 were determined by the area under the receiver operating curves (AUROCs).

Univariate and multivariate logistic regression models were used to investigate the relationship between the SII (continuous and stratified) and the outcome of liver fibrosis. Model 1 was a crude model that was adjusted for none. Model 2 was partially adjusted for sex (female vs. male), age (years), liver cirrhosis (yes vs. no), and ribavirin administration (yes vs. no). Model 3 was a fully adjusted model that included covariates in model 2 and HCV RNA (lg10 IU/L), HCV genotype (vs. 1), baseline total bilirubin (μmol/L), baseline ALT (ULN), baseline albumin (g/L), and baseline serum creatinine (ULN). ALT, AST, and PLT levels measured at PTW12 were not included in Model 3 because of their simultaneous occurrence with the outcome. Baseline AST and PLT levels were not included because their variance inflation factors exceeded 5.

Further stratified logistic regression models were performed with Model 3 to identify variables that modified the relationship between SII (continuous SII and stratified SII) and the outcome of liver fibrosis. Sensitivity analysis using the multiple imputation method was performed. Multiple imputation was used to compensate for missing data based on five replications in the fully conditional specification method to account for missing data on age, total bilirubin, ALT, AST, albumin, globulin, serum creatinine, peripheral white blood cell (WBC), NEU, LYM, and PLT.

Statistical tests were performed using SPSS version 22 (IBM Corp.). Statistical significance was set at a *p*-value of <0.05.

## Results

### Patients’ characteristics

A total of 288 treatment-naive CHC patients treated with an SOF/VEL-based treatment regimen were enrolled and analyzed (Figure 1). Of these patients, the mean age was 49.0 (42.0–56.0) years, 150 (52.1%) were female, 23 (8.0%) had liver cirrhosis, the predominant genotype was 1 and 3, 100 (34.7%) patients had ALT >2 × ULN, 108 (37.5%) patients were given RBV, and 287 (99.7%) patients achieved SVR12 (Table 1).

**Table 1:** Patients’ characteristics.

	Overall (*N* = 288)	SII stratified at 240			SII stratified at 150		
		SII <240 (*n* = 144)	SII ≥240 (*n* = 144)	*p*	SII <150 (*n* = 67)	SII ≥150 (*n* = 221)	*p*
Female	150 (52.1%)	70 (48.6%)	74 (51.4%)	0.238	33 (49.3%)	117 (52.9%)	0.597
Age (years)	49.0 (42.0–56.0)	50.5 (44.0–56.0)	47.5 (36.5–55.0)	0.125	51.0 (44.0–56.0)	49.0 (40.0–55.0)	0.220
Age ≥ 50 years	138 (47.9%)	76 (52.8%)	62 (43.1%)	0.099	37 (55.2%)	101 (45.7%)	0.172
Liver cirrhosis	23 (8.0%)	19 (13.2%)	4 (2.8%)	0.001	9 (13.4%)	14 (6.3%)	0.060
HCV RNA(lg10 IU/L)	6.0 (5.2–6.4)	6.0 (5.3–6.5)	6.0 (5.2–6.5)	0.503	6.0 (5.5–6.4)	6.0 (5.2–6.5)	0.919
HCV genotype				0.905			0.385
1	120 (41.7%)	63 (43.8%)	57 (39.6%)		33 (49.3%)	87 (39.4%)	
2	21 (7.3%)	9 (6.3%)	12 (8.3%)		2 (3.0%)	19 (8.6%)	
3	112 (38.9%)	55 (38.2%)	57 (39.6.2%)		25 (37.3%)	87 (39.4%)	
6	26 (9.0%)	12 (8.3%)	14 (9.7%)		5 (7.5%)	21 (9.5%)	
Unknown	9 (3.1%)	5 (3.5%)	4 (2.8%)		2 (3.0%)	7 (3.2%)	
Total bilirubin (μmol/L)	12.5 (9.5–16.8)	13.2 (9.8–18.3)	11.9 (9.2–15.4)	0.013	12.8 (9.5–18.3)	9.5 (12.4–16.1)	0.795
Alanine aminotransferase (×ULN)	1.4 (0.9–2.4)	1.6 (0.9–2.9)	1.2 (0.8–2.1)	0.007	1.6 (0.8–3.4)	1.3 (0.9–2.4)	0.265
Alanine aminotransferase >2 × ULN	100 (34.7%)	60 (41.7%)	40 (27.8%)	0.013	27 (40.3%)	73 (33.0%)	0.274
Aspartate aminotransferase (×ULN)	50.0 (33.0–79.0)	60.0 (40.3–88.0)	44.5 (31.0–63.0)	0.001	65.0 (36.0–100.0)	48.0 (32.5–69.0)	0.044
Aspartate aminotransferase-to-alanine aminotransferase ratio ≥ 1	89 (30.9%)	51 (35.4%)	38 (26.4%)	0.097	25 (37.3%)	64 (29.0%)	0.195
Albumin (g/L)	44.3 (41.1–47.0)	43.5 (40.1–46.8)	45.1 (42.0–47.2)	0.005	43.5 (36.5–46.5)	44.7 (41.9–47.2)	0.108
Serum creatinine (×ULN)	0.6 (0.5–0.7)	0.6 (0.5–0.7)	0.6 (0.5–0.7)	0.249	0.6 (0.5–0.7)	0.6 (0.5–0.7)	0.484
Platelet (×10^9^/L)	144.0 (103.0–189.5)	106.0 (76.5–148.8)	183.5 (142.0–220.5)	<0.001	85.0 (58.0–105.0)	163.0 (121.5–202.0)	<0.001
Platelet >100 × 10^9^/L	223 (77.4%)	88 (61.1%)	135 (93.8%)	<0.001	22 (32.8%)	201 (91.0%)	<0.001
SII	238.2 (153.0–358.2)	153.3 (104.7–201.3)	357.5 (295.2–462.8)	<0.001	97.8 (72.5–124.4)	289.4 (213.3–391.5)	<0.001
Ribavirin administration	108 (37.5%)	51 (35.4%)	57 (39.6%)	0.465	24 (35.8%)	84 (38.0%)	0.746
SVR12	287 (99.7%)	143 (99.3%)	144 (100.0%)	>0.99	66 (98.5%)	221 (100.0%)	0.233
Outcome of liver fibrosis							
Non-improved APRI	239 (83.0%)	101 (70.1%)	138 (95.8%)	<0.001	35 (52.2%)	204 (92.3%)	<0.001
Non-improved FIB-4	252 (87.5%)	114 (79.2%)	138 (95.8%)	<0.001	50 (74.6%)	202 (91.4%)	<0.001

SII, systemic immune-inflammation index; HCV, hepatitis C virus; ULN, upper limit of normal; SVR12, HCV RNA less than 15 IU/mL 12 weeks after cessation of antiviral therapy; FIB-4, fibrosis-4; APRI, aspartate aminotransferase-to-platelet ratio index. Quantitative data are presented as median (interquartile range) and are compared using Mood’s median test. Qualitative data are presented as frequencies (proportion) and are compared using the chi-squared test.

The median SII was 238.2 (153.0–358.2); 239 (83.0%) and 252 (87.5%) patients had non-improved APRI and FIB-4, respectively (Table 1). The optimal cutoff values of SII at 151.8 and 243.1 for non-improved APRI and FIB-4 were determined by AUROCs. In this study, the SII was stratified at cutoff values of 150 and 240 for non-improved APRI and FIB-4, respectively, for further analysis.

Patients with an SII ≥240 were less likely to have liver cirrhosis (2.8% vs. 13.2%, *p* = 0.001) and ALT >2 × ULN (27.8% vs. 41.7%, *p* = 0.013) (Table 1). Patients with an SII ≥240 were more likely to have non-improved FIB-4 than those with an SII <240 (95.8% vs. 79.2%, *p* < 0.001), and patients with an SII ≥150 were more likely to have non-improved APRI than those with an SII <150 (92.3% vs. 52.2%, *p* < 0.001).

### Association between SII and outcome of liver fibrosis

**Figure 2:**
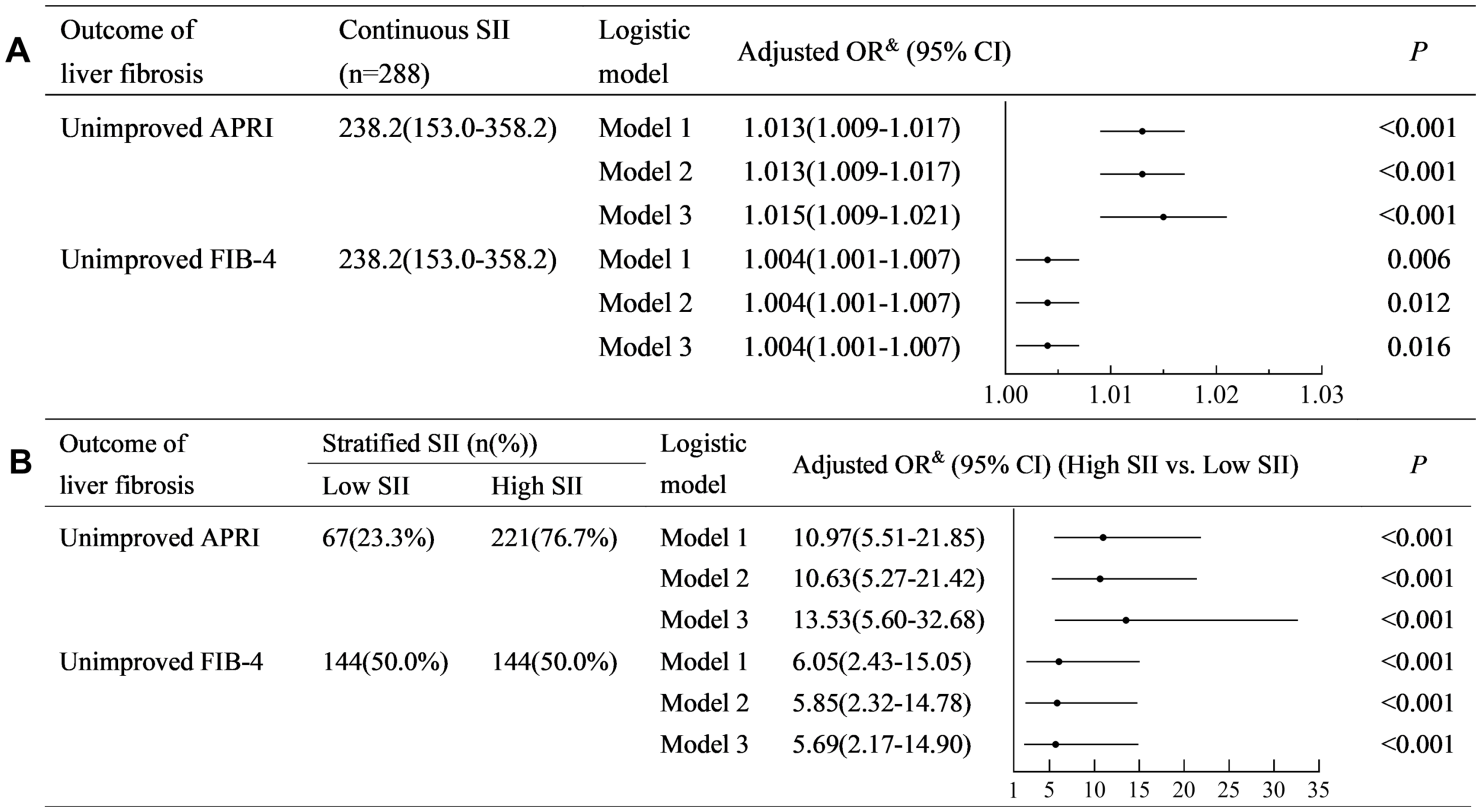
Univariate and multivariate logistic regression analyses to identify the association between SII and the outcome of liver fibrosis. **(A)** Analysis with continuous SII; **(B)** Analysis with stratified SII. SII, systemic immune-inflammation index; FIB-4, fibrosis-4; APRI, aspartate aminotransferase-to-platelet ratio index; OR, odds ratio; CI, confidence interval. Model 1: a crude model adjusted for none. Model 2: a partially adjusted model including sex (female vs. male), age (years), liver cirrhosis (yes vs. no), and ribavirin administration (yes vs. no). Model 3: a fully adjusted model including covariates in model 2 and HCV RNA(lg10 IU/L), HCV genotype (vs. 1), baseline total bilirubin (μmol/L), baseline alanine aminotransferase (×ULN), albumin (g/L), and baseline serum creatinine (×ULN).

The SII was positively associated with non-improved APRI (Model 3: adjusted odds ratio (OR) (95% confidence interval (CI)): 1.015 (1.009–1.017), *p* < 0.001) (Figure 2A). Similarly, SII was positively associated with non-improved FIB-4 (Model 3: adjusted OR (95% CI): 1.004 (1.001–1.007), *p* < 0.001).

A higher SII (≥150) was associated with a higher risk of non-improved APRI (Model 3: adjusted OR (95% CI): 13.53 (5.60–32.68), *p* < 0.001) (Figure 2B). Similarly, a higher SII (≥240) was associated with a higher risk of non-improved FIB-4 (Model 3: adjusted OR (95% CI): 5.69 (2.17–14.90), *p* < 0.001).

### Potential factors modifying the association between SII and the outcome of liver fibrosis

**Figure 3:**
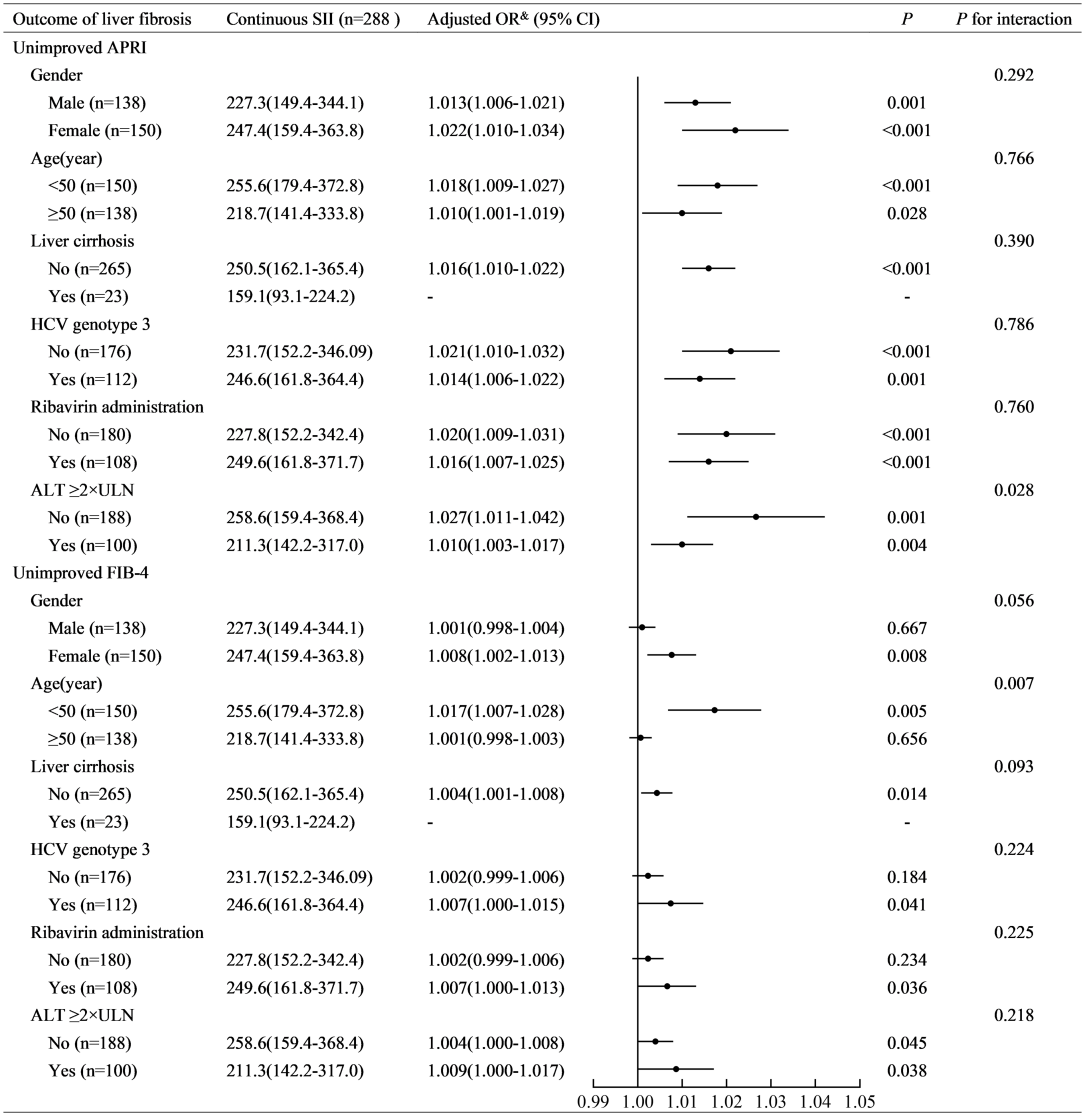
Stratified logistic regression analysis to identify variables that modify the correlation between continuous SII and the outcome of liver fibrosis. SII, systemic immune-inflammation index; APRI, aspartate aminotransferase-to-platelet ratio index; FIB-4, fibrosis-4; ALT, alanine aminotransferase; ULN, upper limit of normal value; OR, odds ratio; CI, confidence interval. Adjusted OR^&^: a fully adjusted model (Model 3) adjusted for sex (female vs. male), age (years), liver cirrhosis (yes vs. no), ribavirin administration (yes vs. no), HCV RNA(lg10 IU/L), HCV genotype (vs. 1), baseline total bilirubin (μmol/L), baseline alanine aminotransferase (×ULN), baseline albumin (g/L), and baseline serum creatinine (×ULN).

**Figure 4:**
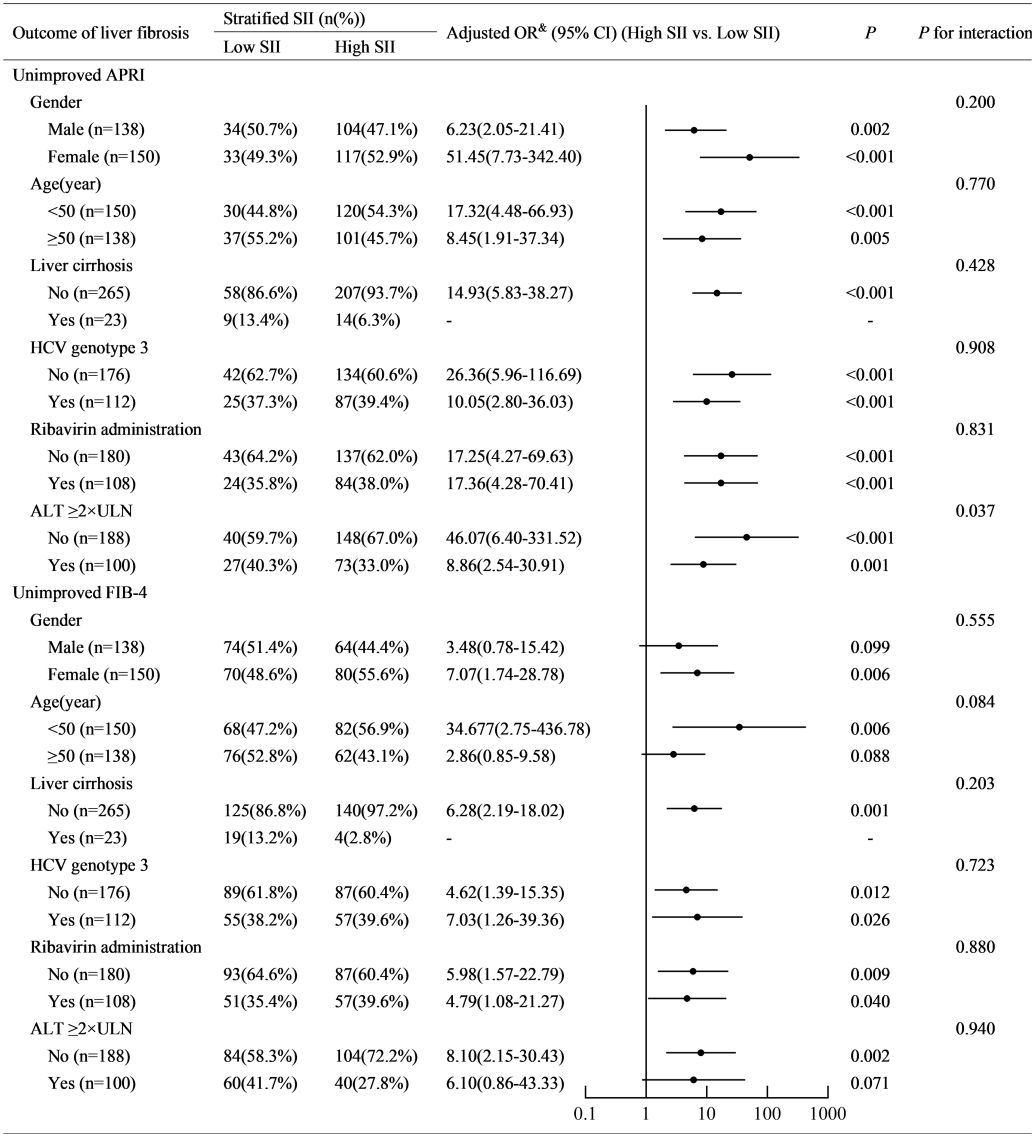
Stratified logistic regression analysis to identify variables that modify the correlation between stratified SII and the outcome of liver fibrosis. SII, systemic immune-inflammation index; APRI, aspartate aminotransferase-to-platelet ratio index; FIB-4, fibrosis-4; ALT, alanine aminotransferase; ULN, upper limit of normal value; OR, odds ratio; CI, confidence interval. Adjusted OR^&^: a fully adjusted model (Model 3) adjusted for sex (female vs. male), age (years), liver cirrhosis (yes vs. no), ribavirin administration (yes vs. no), HCV RNA(lg10 IU/L), HCV genotype (vs. 1), baseline total bilirubin (μmol/L), baseline alanine aminotransferase (×ULN), baseline albumin (g/L), and baseline serum creatinine (×ULN).

SII was positively associated with non-improved APRI in patients with ALT <2ULN (adjusted OR (95% CI): 1.027 (1.011–1.042), *p* = 0.001) and those with ALT ≥2ULN (adjusted OR (95% CI): 1.010 (1.003–1.017), *p* = 0.004) (Figure 3). A higher SII (≥150) was associated with a higher risk of non-improved APRI in patients with ALT <2ULN (adjusted OR (95% CI): 46.07 (6.40–331.52), *p* < 0.001) and those with ALT ≥2ULN (adjusted OR (95% CI): 8.86 (2.54–30.91), *p* = 0.001) (Figure 4). Stratified multivariate logistic regression analysis (Model 3) demonstrated that ALT was a potential modifier in the relationship between SII and non-improved APRI (*P* for interaction =0.028) and in the relationship between stratified SII (high SII vs. low SII) and non-improved APRI (*P* for interaction =0.037).

Similarly, SII was positively associated with non-improved FIB-4 in patients aged <50 years (adjusted OR (95% CI): 1.017 (1.007–1.028), *p* = 0.005). However, it was not similar to that in patients aged ≥50 years (adjusted OR (95% CI): 1.001 (0.998–1.003), *p* = 0.656) (Figure 3). Stratified multivariate logistic regression analysis (Model 3) demonstrated that age was a potential modifier in the relationship between SII and non-improved FIB-4 (*P* for interaction =0.007). No potential modifiers were found in the relationship between the stratified SII (high SII vs. low SII) and non-improved FIB-4 (all *P* for interaction >0.05).

### Sensitivity analysis of the association between SII and the outcome of liver fibrosis

**Figure 5:**
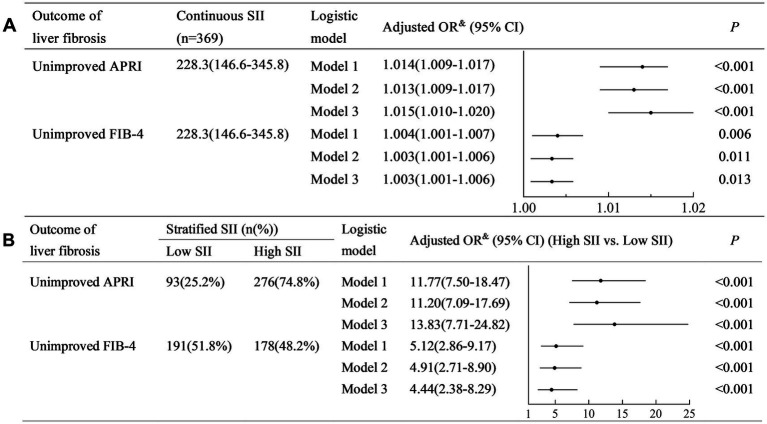
Multiple imputation analysis for the detection of sensitivity. **(A)** Analysis with continuous SII; **(B)** Analysis with stratified SII. Model 1: a crude model adjusted for none. Model 2: a partially adjusted model including sex (female vs. male), age (years), liver cirrhosis (yes vs. no), and ribavirin administration (yes vs. no). Model 3: a fully adjusted model including covariates in model 2 and HCV RNA(lg10 IU/L), HCV genotype (vs. 1), baseline total bilirubin (μmol/L), baseline alanine aminotransferase (×ULN), baseline albumin (g/L), and baseline serum creatinine (×ULN).

Multiple imputation was performed to validate our results. The distribution of patient characteristics is shown in Table 2. Sensitivity analysis demonstrated similar results that the SII was positively associated with non-improved APRI and FIB-4 in Models 1–3 (all crude and adjusted OR > 1.00, all *p* < 0.05) (Figure 5A), and a higher SII was associated with a higher risk of non-improved APRI and FIB-4 in Models 1–3 (all crude and adjusted OR > 1.00, all *p* < 0.001) (Figure 5B).

**Table 2:** Distributions of patients’ characteristics among complete cases and multiple imputation.

	Complete case (*N* = 288)	Multiple imputation (*N* = 369)
Female	150 (52.1%)	188 (50.9%)
Age (years)	49.0 (42.0–56.0)	49.0 (42.0–56.0)
Age ≥ 50 years	138 (47.9%)	181 (49.1%)
Liver cirrhosis	23 (8.0%)	36 (9.8%)
HCV RNA(lg10 IU/L)	6.0 (5.2–6.4)	5.9 (5.2–6.5)
HCV Genotype		
1	120 (41.7%)	154 (41.7%)
2	21 (7.3%)	28 (7.6%)
3	112 (38.9%)	138 (37.4%)
6	26 (9.0%)	37 (10.0%)
Unknown	9 (3.1%)	12 (3.3%)
Total bilirubin (μmol/L)	12.5 (9.5–16.8)	12.6 (9.4–17.2)
Alanine aminotransferase (×ULN)	1.4 (0.9–2.4)	1.4 (0.9–2.5)
Alanine aminotransferase >2 × ULN	100 (34.7%)	130 (35.2%)
Albumin (g/L)	44.3 (41.1–47.0)	44.2 (40.8–46.8)
Serum creatinine (×ULN)	0.6 (0.5–0.7)	0.6 (0.5–0.7)
Platelet (×10^9^/L)	144.0 (103.0–189.5)	144.0 (100.5–189.0)
Platelet >100 × 10^9^/L	223 (77.4%)	277 (75.1%)
SII	238.2 (153.0–358.2)	228.3 (146.6–345.8)
Ribavirin administration	108 (37.5%)	135 (36.6%)
SVR12	287 (99.7%)	366 (99.2%)
Outcome of liver fibrosis		
Non-improved APRI	239 (83.0%)	299 (81.0%)
Non-improved FIB-4	252 (87.5%)	323 (87.5%)

SII, systemic immune-inflammation index; HCV, hepatitis C virus; ULN, upper limit of normal; SVR12, HCV RNA less than 15 IU/mL 12 weeks after cessation of antiviral therapy; FIB-4, fibrosis-4; APRI, aspartate aminotransferase-to-platelet ratio index. Quantitative data are presented as median (interquartile range). Qualitative data are presented as frequencies (proportions).

## Discussion

DAAs have dramatically changed the landscape of hepatitis C treatment and prevention. The World Health Organization (WHO) has called for the elimination of hepatitis C as a public health threat by 2030. However, the discrepancy in DAA prices across low-, middle-, and high-income countries is considerable, thus representing a major barrier to the scale-up of treatment and elimination (26). In China, HCV elimination is no longer a challenge because of the availability and affordability of DAAs, facilitated by the mass production of domestic pharmaceuticals and the implementation of medical insurance reimbursement schemes (27). Future management will concentrate on broadening screening, linking to treatment, and surveillance of decompensated cirrhosis and HCC after SVR (27, 28). Multiple studies have emphasized the non-negligible incidence of deterioration of liver stiffness or HCC in patients’ post-treatment, especially in patients with F3/F4 fibrosis. “Risk-based surveillance strategies” are now under establishment (29). Liver stiffness is the two main mechanisms underlying the persistence of residual HCC after HCV eradication. Advanced fibrosis or cirrhosis is difficult to resolve because of the inability to remodel after HCV eradication, at which time the risk of HCC remains. The other one believes that preneoplastic genetic/epigenetic changes or monoclonal micronodules that already exist during hepatotoxic injuries to fibrosis could persist “indefinitely” after SVR (30). Therefore, an optimal indicator to predict improvement in liver stiffness after DAA treatment would contribute to establishing a surveillance strategy for long-term prognosis.

An Italian study by Bologna et al. reported that, combined with the albumin–bilirubin score, immune-inflammation indicators could help identify the occurrence or recurrence of HCC in patients after DAA treatment (31). This implies that immune-inflammatory indicators are suitable for long-term surveillance. SII integrates PLT, NEU, and LYM to reflect the local immune response and systemic inflammation in the entire human body. A high SII is a poor prognostic factor for multiple cancers. A meta-analysis of 22 studies with 7,657 patients with cancer showed that a higher level of SII was correlated with poor overall survival (HR: 1.69, 95% CI: 1.42–2.01, *p* < 0.001) and poor recurrence-free survival (HR: 1.66, 95% CI: 1.07–2.59, *p* = 0.025) (32). However, whether SII levels influence the prognosis of patients with liver diseases and their correlation with liver stiffness has not been addressed.

Our study adds to this concern through further investigation. In this secondary data analysis based on a prospective real-world study, we found that SII was positively associated with non-improved outcomes of liver fibrosis assessed with APRI (non-improved APRI) and FIB-4 (non-improved FIB-4) in treatment-naive patients with CHC who received SOF/VEL-based therapy. A higher SII (≥150 and ≥ 240) was associated with a higher risk of non-improved APRI and FIB-4, respectively. A high SII also exhibits excellent prognostic value in HCC. Vascular invasion, large tumors, or early recurrence was more frequent when the SII was ≥330 (7). In addition, the number of circulating tumor cells was significantly higher in the SII ≥330 group (7). These findings further supplement the evidence that the SII is an optimal predictor for prognosis to some degree as there are plenty of research studies clarifying the modulated hepatocarcinogenesis from fibrosis to HCC. For example, increased liver stiffness induces loss of tensional homeostasis, which increases cancer risk and accelerates tumor progression (33). Moreover, enriched HSC in the preneoplastic environment would closely interact with hepatocytes, regulating their proliferation and apoptosis (34).

It is worth noting that the association between the SII or stratified SII and non-improved APRI was much more remarkable in patients with ALT <2 ULN. A possible reason is that in patients with hepatitis and normal or moderately elevated ALT levels, APRI could not accurately reflect the actual degree of liver stiffness (sensitivity = 42.39%) (35). The SII can reflect reality and be a better predictor in such patients. Another hypothesis is that in patients with advanced fibrosis, normal follow-up ALT levels (<40 IU/L) are negatively related to histological progression (36). Patients with ALT <2 ULN had a higher chance of maintaining ALT levels <40 IU/L. Nevertheless, these results warrant further investigation. Moreover, the association between the SII and non-improved FIB-4 was remarkable in patients aged <50 years. The mechanism may underlie the stronger neutrophil phagocytic and bactericidal effects in young and middle-aged patients, which is believed to provide the “fuel that feeds the flames” (37). HSC can be better activated by enhanced flames to accelerate fibrogenesis.

Interestingly, although a positive relationship to non-improved liver fibrosis in CHC patients post-DAA was found in our study, another study reported the reverse predictive effects of inflammatory scores in patients with BCLC 0-A HCC after hepatectomy. A low SII (≤160) was found to be an independent risk factor for poor overall survival. They assumed that these results were due to thrombocytopenia as there was no significant difference in the non-thrombocytopenia subgroup (38). Low PLT mostly refers to advanced liver disease, while thrombocytopenia was also found to be an independent predictor of survival in both compensated cirrhotic patients and post-HCC resection patients (39). In our study, the median PLT count was 144.0 (103.0–189.5) × 10^9^/L. The fact that most patients (77.4%) had normal PLT levels (>100 × 10^9^/L) may have resulted in this difference. The baseline inflammatory status also helps explain this finding. Cirrhosis-associated immune dysfunction is dynamic. In the end-stage state, host immune-inflammation is exhausted (40). Both local and systemic immune-inflammation were impaired at that time. A similar phenomenon was observed in a population-based study that analyzed SII levels in patients with non-alcoholic fatty liver disease. They found a positive relationship between increased SII and advanced hepatic steatosis but not liver fibrosis (22). Baseline ALT in that study was normal, while it was elevated to 1.4 (0.9–2.4) × ULN in our cohort.

This study has some limitations. First, the patients originated from a single geographic region, with a considerable amount of missing data. These factors could affect the generalizability and robustness of our findings. Second, the patients in our study were all treatment-naive patients without HBV or HIV infection and decompensated liver cirrhosis. The results derived from this subset may not apply to patients with these characteristics. Third, the follow-up time was short, and serological indicators were used to evaluate liver fibrosis and its outcomes. The observed decline in non-invasive assessment of liver fibrosis might be the result of a combination of factors, including reversal of liver fibrosis (41–43), improvement in hepatocellular inflammation (43–45), normalization of ALT (45, 46), increase in PLT (47–49), and random errors in laboratory testing. In addition, the cutoff values for FIB-4 and APRI to assess liver fibrosis were established based on treatment-naive patients (24, 25), while the corresponding values for non-invasive assessment of liver fibrosis for treatment-experienced patients remain to be elucidated (45). In this study, the use of FIB-4 and APRI instead of liver biopsies and the use of cutoff values established based on treatment-naive patients might have affected the results of the study.

In conclusion, cirrhosis occurs in patients with HCV infection even after virus elimination by DAAs. However, an optimal predictor of long-term prognosis is lacking. Our findings suggested that the SII was positively associated with non-improved outcomes of liver fibrosis in patients with CHC. More attention should be paid to patients with a high SII because of their poor prognosis. Further large-scale, multicenter, prospective cohort studies are warranted to validate the performance of the SII. A validated model with SII would help to assess disease severity and predict outcomes in patients with CHC post-treatment and guide clinical management.

## Data Availability

The original contributions presented in the study are included in the article/supplementary material, further inquiries can be directed to the corresponding authors.

## Glossary

ALT: alanine aminotransferase
APRI: aspartate aminotransferase-to-platelet ratio index
AST: aspartate aminotransferase
AUROCs: area under the receiver operating curves
CHC: chronic hepatitis C
CI: confidence interval
COVID-19: coronavirus disease 2019
CT: computed tomography
DAAs: directly acting antivirals
FIB-4: fibrosis-4 index
HBV: hepatitis B virus
HCC: hepatocellular carcinoma
HCV: hepatitis C virus
HIV: human immunodeficiency virus
HSC: hepatic stellate cell
LYM: lymphocyte
NEU: neutrophil
OR: odds ratio
PLT: platelet
RBV: ribavirin
SII: systemic immune-inflammatory index
SOF/VEL: sofosbuvir/velpatasvir
SVR: sustained virologic response
ULN: upper limit of normal
